# Exposure to PM_2.5_ and Obesity Prevalence in the Greater Mexico City Area

**DOI:** 10.3390/ijerph18052301

**Published:** 2021-02-26

**Authors:** Marcela Tamayo-Ortiz, Martha María Téllez-Rojo, Stephen J. Rothenberg, Ivan Gutiérrez-Avila, Allan Carpenter Just, Itai Kloog, José Luis Texcalac-Sangrador, Martin Romero-Martinez, Luis F. Bautista-Arredondo, Joel Schwartz, Robert O. Wright, Horacio Riojas-Rodriguez

**Affiliations:** 1 Occupational Health Research Unit, Mexican Institute of Social Security, 06720 Mexico City, Mexico; tamayo.marcela@gmail.com; 2 National Institute of Public Health, 62100 Cuernavaca, Mexico; drlead@prodigy.net.mx (S.J.R.); ivan_2c@hotmail.com (I.G.-A.); jtexcalac@insp.mx (J.L.T.-S.); martin.romero@insp.mx (M.R.-M.); lbautista@insp.mx (L.F.B.-A.); hriojas@insp.mx (H.R.-R.); 3 Department of Environmental Medicine and Public Health, Icahn School of Medicine at Mount Sinai, New York, NY 10029, USA; allan.just@mssm.edu (A.C.J.); robert.wright@mssm.edu (R.O.W.); 4 Department of Geography and Environmental Development, Ben-Gurion University of the Negev, Beer-Sheva 8410501, Israel; itai.kloog@mssm.edu; 5 Department of Epidemiology, Harvard T.H. Chan School of Public Health, Boston, MA 02115, USA; joel@hsph.harvard.edu

**Keywords:** PM_2.5_ exposure, obesity, Mexico, Latin America

## Abstract

Exposure to PM_2.5_ has been associated with the prevalence of obesity. In the Greater Mexico City Area (GMCA), both are ranked among the highest in the world. Our aim was to analyze this association in children, adolescents, and adults in the GMCA. We used data from the 2006 and 2012 Mexican National Surveys of Health and Nutrition (ENSANUT). Participants’ past-year exposure to ambient PM_2.5_ was assessed using land use terms and satellite-derived aerosol optical depth estimates; weight and height were measured. We used survey-adjusted logistic regression models to estimate the odds ratios (ORs) of obesity (vs. normal-overweight) for every 10 µg/m^3^ increase in annual PM_2.5_ exposure for children, adolescents, and adults. Using a meta-analysis approach, we estimated the overall odds of obesity. We analyzed data representing 19.3 million and 20.9 million GMCA individuals from ENSANUT 2006 and 2012, respectively. The overall pooled estimate between PM_2.5_ exposure and obesity was OR = 1.96 (95% CI: 1.21, 3.18). For adolescents, a 10 µg/m^3^ increase in PM_2.5_ was associated with an OR of 3.53 (95% CI: 1.45, 8.58) and 3.79 (95% CI: 1.40, 10.24) in 2006 and 2012, respectively. More studies such as this are recommended in Latin American cities with similar air pollution and obesity conditions.

## 1. Introduction

Expanding the epidemiologic evidence linking PM_2.5_ exposure to adverse health outcomes is key for advancing public health policies and regulations to improve air quality and preventing disease worldwide, such as the obesity epidemic. Between 1975 and 2014, the prevalence of obesity in adults worldwide increased from 3.2% (2.4–4.1) to 10.8% (9.7–12.0) for men, and from 6.4% (5.1–7.8) to 14.9% (13.6–16.1) in women [1]. Once considered a high-income country problem, we now know that obesity affects low- and middle-income countries equally [2], and it is becoming more prevalent at younger ages. According to the Mexican National Survey of Health and Nutrition (ENSANUT), in 2018, the combined prevalence of overweight and obesity in children (5 to 11 years-old), adolescents (12 to 19 years-old), and adults (≥20 years-old) were 35.6%, 38.4%, and 75.2%, respectively [3].

The Greater Mexico City Area (GMCA), one of the largest and most populated cities in Latin America, is home to over 21 million people, where PM_2.5_ concentrations frequently exceed the WHO air quality guidelines for short-term (24-h standard average, GMCA = 96 µg/m^3^ vs. WHO < 25 µg/m^3^) and chronic (annual average GMCA = 21 µg/m^3^ vs. WHO =10 µg/m^3^) exposure [4], and where the combined prevalence for overweight and obesity mimics the national prevalence for children, adolescents, and adults [5]. Existing studies on air pollution (not specifically for PM_2.5_) have diverse findings, suggesting further research in developing countries and heterogenous populations [6].

One of the proposed mechanisms linking PM_2.5_ exposure to obesity involves Toll-like receptors in the lung, which through inflammation and lipid oxidation can lead to metabolic dysfunction and weight gain [7]. A recent study in mice has shown that chronic hypothalamic inflammation, regulated by signaling of these Toll-like receptors, can lead to leptin resistance, hyperphagia, and decreased energy expenditure [8]. This is line with results from other animal studies that have shown that early life exposure to PM_2.5_ leads to the development of insulin resistance, adiposity, and inflammation [9]. Prenatal exposure to PM_2.5_ has also been linked to increased insulin levels and glucose intolerance [10]. Moreover, effects can be sex-specific [11]. Exposure during animal adulthood has been found to induce obesity, in part because of less energy expenditure (due to hypothalamic leptin resistance) [10]. Nevertheless, results from human studies on the association between PM_2.5_ exposure and obesity remain mixed [12]. Some have found null associations [13,14], while several others point to an increased risk of obesity from PM_2.5_ exposure at different life stages [15,16,17,18,19,20,21,22,23,24,25,26,27,28,29,30,31]. Therefore, the aim of this study was to analyze the association between past-year exposure to PM_2.5_ with the prevalence of obesity in two representative samples (ENSANUT 2006 and 2012) of children, adolescents, and adults from the GMCA.

## 2. Materials and Methods

### 2.1. Study Population

We used data from the 2006 and 2012 ENSANUT surveys that use a multi-stage and stratified clustered sampling procedure at the household level and are designed to be nationally and state-representative [32]. Both 2006 and 2012 surveys included overweight and obesity data for children (2–9 years-old), adolescents (10–19 years-old), and adults (≥20 years-old).

Our study used sample weights to ensure the sample representativeness for the 16 municipalities of Mexico City and 58 municipalities from the State of Mexico that comprise the GMCA. The Ethics Committee of the Mexican National Institute of Public Health (INSP) reviewed and approved the written consent forms employed in ENSANUT 2006 and 2012, as well as our research protocol.

### 2.2. PM_2.5_ Exposure Assessment

Geolocation of households was estimated at a block level; no further detail was pursued in order to maintain participant’s confidentiality. We used daily PM_2.5_ estimates from a hybrid spatio-temporal model developed by colleagues in our research team that has been previously described in more detail [33]. Briefly, the model estimates daily PM_2.5_ concentrations with a spatial resolution of 1 × 1 km^2^ for the entire GMCA by calibrating the association between PM_2.5_ measurements from ground monitors to satellite-derived aerosol optical depth measurements from the United States’ National Aeronautics and Space Administration (NASA), including land use terms and meteorological features specific to the GMCA. Daily exposure for each household was calculated from the 1 × 1 km^2^ PM_2.5_ cells, matching each household’s block area. We calculated the average past-year PM_2.5_ exposure according to the date the survey was administered.

### 2.3. Anthropometric Assessment

All participants were weighed twice (and averaged) using calibrated portable electronic scales (Tanita, Model 1583, Tokyo, Japan) recording the measurement to the nearest 10 g. Height was measured twice (and averaged) in orthostatic position using a stadiometer (Dynatop E1, Mexico City, Mexico) with a precision of 1 mm. Both anthropometric measures were performed by trained staff using standardized methods. We used these measurements to calculate body mass index (BMI) as the ratio of weight to height squared (Kg/m^2^). To determine participants’ overweight (25 ≤ BMI < 30) and obesity (BMI ≥ 30) status in adults, we followed the cut-off points in the WHO guidelines, and for children and adolescents, we followed age-specific guidelines from the Mexican Social Security Institute (IMSS) that follow age-specific WHO guidelines.

### 2.4. Covariates

Information on sex, age, smoking status (adolescents and adults), second-hand smoke (children), and socioeconomic status (SES) was collected by questionnaire during the survey home visit. SES was calculated using information on income levels and demographic and socioeconomic characteristics of Mexican households on the basis of the National Survey of Household Income and Expenditure [34]. For the adolescent and adult surveys, there was no information on second-hand smoking available. We considered non-smoking for individuals reporting never smoking vs. individuals that reported any smoking. For children, spending money on tobacco was used as a proxy for second-hand smoke. The same questions were asked in the 2006 and 2012 surveys. The field staff was trained and standardized previous to field work by a team of researchers from the National Institute of Public Health (INSP). 

### 2.5. Statistical Analysis

We used Stata V.14 “svy” package for survey analysis (Stata Corp, College Station, TX, USA) to fit logistic regression models incorporating the sample design in the variance estimates. We estimated the association between the average past-year PM_2.5_ exposure and obesity prevalence and calculated Taylor linearized standard errors. Models were adjusted by sex, age in quintiles, SES, and smoking status. For this first analysis, we collapsed normal and overweight into one category and used it as the reference to compare with obesity. For each age group (i.e., children, adolescents, and adults), we performed separate models for each survey year (i.e., 2006, 2012). PM_2.5_ exposure estimates were included as continuous variables in our main analysis. In separate models, we included an interaction term with sex and then stratified by sex. We present adjusted odds ratios (ORs) for an increase of 10 μg/m^3^ in PM_2.5_. We also generated a multinomial regression model to evaluate overweight and obesity simultaneously compared to normal weight subjects and obtained relative risk ratios (RRR). To assess the non-linearity of the associations, we used quartiles of PM_2.5_ with a logistic model comparing normal and overweight to obesity. Lastly, we used a meta-analysis, random effect approach to estimate the overall association between PM_2.5_, and obesity (i.e., obesity vs. normal/overweight) using the combined results from all age groups from the 2006 and 2012 analyses and by age group.

Additionally, in order to exclude the possible measurement error due to smoking or second-hand smoke, we conducted the main analysis considering non-smoker adolescents and adults and children with no house-hold tobacco spending.

## 3. Results

A total of 4368 and 4521 persons participated in the ENSANUT 2006 and 2012 surveys representing 19.4 and 21 million inhabitants of the GMCA, respectively. Sex ratios were approximately 1:1. The mean age (±SD) of children, adolescents, and adults were similar in both survey years. While the prevalence of normal weight was similar between surveys, we saw a decrease in overweight and an increase in obesity prevalence for all age groups in the 2012 sample. The average past-year PM_2.5_ concentrations showed a slight decrease from 2006 to 2012 (Table 1).

Overall, exposure to PM_2.5_ was associated with higher odds of obesity in each survey and all age groups (Table 2), with stronger results for adolescents. For every 10 μg/m^3^ increase in PM_2.5_, the odds of obesity were 3.53 (95% CI: 1.45, 8.58) in 2006 and 3.79 (95% CI: 1.40, 10.24) in 2012. The results for the 2006 survey for children and adults also showed increased odds for obesity OR = 1.19 (95% CI: 0.47, 3.08) and OR = 1.01 (95% CI: 0.59, 1.73), respectively, and the results for children and adults in the 2012 survey showed increased odds of 1.98 (95% CI: 0.92, 4.22) and 2.73 (95% CI: 0.97, 7.71), respectively.

Only the results for the 2006 survey in children showed an interaction term between PM_2.5_ and sex (*p* for interaction = 0.07). There was no consistency in the models stratified by sex—while in the 2006 results, the effect of PM_2.5_ on obesity was stronger in male adolescents than in females (OR_male_ = 4.19 (95% CI 1.24, 14.19)), it was stronger for females in 2012 (OR_female_ = 7.83 (95% CI 0.96, 64.12)). We found similar inconsistencies by sex in children and adults between surveys.

The results for adolescents in the multinomial models were in line with the logistic models showing a RRR_overweight_ of 1.36 (95% CI 0.68, 2.73) and RRR_obesity_ of 3.89 (95% CI 1.51, 10.03) compared to normal weight in 2006 and a RRR_overweight_ of 2.34 (95% CI 0.83, 6.65) and RRR_obesity_ of 4.82 (95% CI 2.16, 10.76) compared to normal weight in 2012 (Table 2 and Table 3). For children, the RRR for obesity were also higher than for overweight compared to normal weight in both surveys. For adults, the 2012 results showed a higher RRR for obesity than for overweight compared to normal weight.

**Figure 1:**
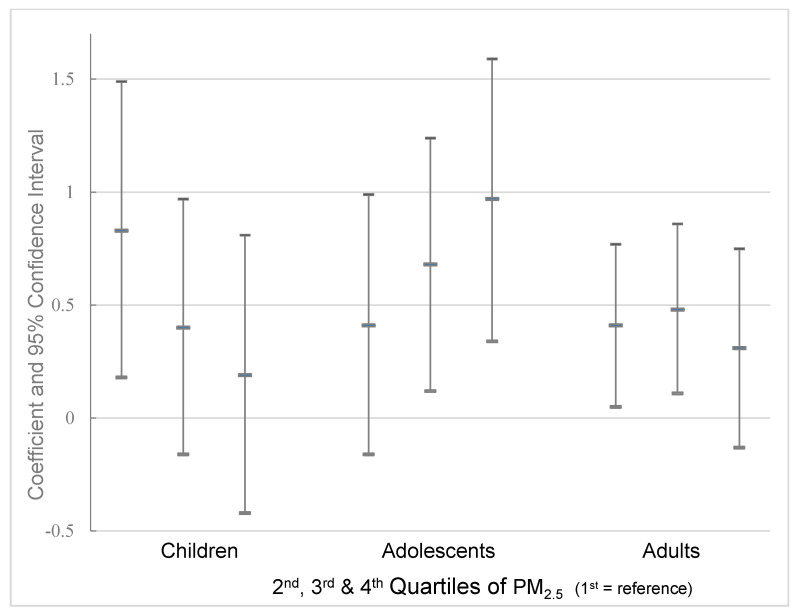
Associations between quartiles of PM_2.5_ (annual mean) and obesity in three age groups, ENSANUT 2012.

Associations from the models using PM_2.5_ in quartiles (with the first quartile of PM_2.5_ as a reference) are shown for the 2012 survey in Figure 1. Estimates of the model for adolescents showed an increasing dose–response relation as follows: second quartile ß = 0.41 (95% CI: −0.16, 0.99), third quartile ß = 0.68 (95% CI: 0.12, 1.24), and fourth quartile ß = 0.97 (95% CI: 0.34, 1.59). In children, an inverse relation was observed. Estimates were ß = 0.83 (95% CI: 0.18, 1.49) for the second quartile, ß = 0.40 (95% CI: −0.16, 0.97) for the third quartile, and ß = 0.19 (95% CI: −0.42, 0.81) for the fourth quartile (first quartile of PM_2.5_ as a reference). For adults, the estimates showed an increasing relation for the second and third quartiles at ß = 0.41 (95% CI: 0.05–0.77) and ß = 0.48 (95% CI: 0.11–0.86), respectively, but this did not hold for the fourth quartile at ß = 0.31 (95% CI: −0.13, 0.75) (first quartile of PM_2.5_ as reference).

**Figure 2:**
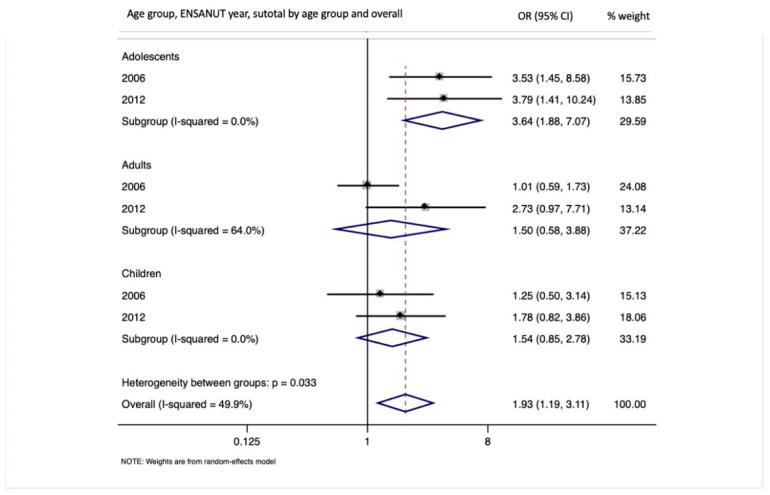
Odds ratios for obesity and PM_2.5_ exposure (per 10 µg/m^3^ increase) using a meta-analysis approach, by age group and for the overall study.

Figure 2 shows the combined results of the meta-analysis with an overall OR of 1.96 (95% CI: 1.21, 3.18) for the association between PM_2.5_ exposure and obesity. When analyzing by age group, we found that the combined OR for adolescents was 3.64 (95% CI: 1.88, 7.06), OR = 1.50 (95% CI: 0.58, 3.88) for adults, and OR = 1.62 (95% CI: 0.90, 2.93) for children. Most of the heterogeneity in the combined estimate was attributable to adults between the two surveys.

In the analyses considering non-smokers (adults and adolescents) and no second-hand smoke (children), the odds for obesity in children were lower for both study years, whereas for adolescents and adults, the odds increased in comparison to the model with all participants. The meta-analysis showed an overall increase in the odds of the association, OR = 2.27 (95% CI: 1.08, 4.75) (data not shown).

## 4. Discussion

Our study analyzed the association between average past-year satellite-derived exposure to PM_2.5_ and the prevalence of obesity in of children, adolescents, and adults at two time points. This approach is different from other studies that have focused on a single age group or a specific population sample; furthermore, this is the first study with a representative population in one of the largest and most populated cities in Latin America. Overall, we observed an almost twofold increase in the odds of obesity (OR_pooled_ = 1.96 (95% CI: 1.21, 3.18)) for each 10 µg/m^3^ of PM_2.5_. However, the association was strongest for adolescents, in line with other studies that have found, that exposure to high levels of traffic density was associated with higher attained BMI over an eight-year follow-up, and that traffic related air pollution had a stronger association compared to traffic density [25,28]. There is also evidence of obesity exacerbating the effects of air pollution on cardiometabolic disease markers [35]. A study in non-diabetic Indonesian adolescents found an association of long-term PM_2.5_ exposure and an increase in fasting plasma glucose levels [26], adding to the knowledge of possible mechanisms related to the association we observed in this age group. Although the results for children in the 2012 survey were marginally significant, those for 2006 were not. Nevertheless, both showed increased odds of obesity, similar to other cross-sectional studies in China and Spain that have found increased risk of obesity with PM_10_ and PM_2.5_, respectively [18,19]. Longitudinal studies in children that have assessed air pollution exposure since early stages (some including prenatal exposures) have mixed findings, with positive [23], null [13,36], and inverse associations [15]. Furthermore, the association for prenatal exposure to PM_2.5_ and childhood obesity seems to be stronger for those born to obese mothers [31]. Our study was unable to account for earlier life or prenatal PM_2.5_ exposure, but assuming there was no strong variability of PM_2.5_ between 2006 and 2012, our results suggest that there might be an increased risk for obesity as the population transitions from childhood to adolescence. Then, in the transition from adolescence to adulthood, unmeasured confounders (such as diet and physical activity) might be driving the apparent reduction in risk we observed.

Our results for adults were inconsistent from 2006 to 2012, although evidence has also pointed at an increased risk of obesity in this age group [22]. Many adult studies [27,37] and reviews [38,39] have focused on the association of PM_2.5_ exposure and increased susceptibility to cardiovascular diseases and type 2 diabetes in adults with obesity. PM_2.5_ might be associated more directly with metabolic disorders in adults, rather than with obesity directly through oxidative stress and inflammation [37]. We found no consistent evidence of an interaction between PM_2.5_ and sex in any age group or across the time points, which is different to other studies that have found that women have a higher risk of obesity associated with exposure to intensive traffic [20].

We were able to include SES in our study, for which there is extensive research as an important predictor of both obesity [40,41] as well as of PM _2.5_ exposure [42,43], therefore possibly confounding the association. In our study, adjusted models for each age group and survey year confirmed the association between lower SES and higher PM_2.5_ as well as increased SES and lower odds of obesity. The effect estimates of our models reflected an important change when adjusting for SES. For all age groups and years, increased SES was associated with reduced odds of obesity (reaching statistical significance only for children 2006 *p* < 0.05 and adolescents 2006 *p* = 0.05), except for adults in 2012 where increased SES was associated with and increased odds of obesity (OR 1.14; 95% CI: 0.99, 1.32).

Smoking status is an important factor to consider, since households with tobacco smoke exposure can be so overwhelming that it can mask the effects of outdoor exposure in its association with obesity [44], and although our main analyses were adjusted for smoking status and second-hand smoke for children, we investigated how our effect estimates would change by including only non-smokers and no second-hand smoke in children’s household. This strengthened the effect estimates for adolescents and adults as well as the overall association (data not shown). However, we could not account for second-hand smoke for adults or adolescents, which could be a different comparison than what we included in this analysis.

In terms of biological mechanisms, animal studies have shown associations between PM_2.5_ exposure and an increase in inflammation and adiposity [9,45,46], changes in energy metabolism [47], and alterations in food intake and dietary behaviors increasing the risk of obesity [11,48]. It has been also documented that that TLR2/4-dependent inflammatory activation and lipid oxidation in the lung triggered by PM_2.5_ exposure can spill over systemically, leading to metabolic dysfunction and weight gain [7], as well as a transgenerational transmission of obesity developmental programming [46]. Another recent animal study suggests suppressing oxidative stress and inflammatory response in order to prevent and treat air pollution-induced diseases such a non-alcoholic fatty liver disease [8]. A study in Mexico City demonstrated possible mechanisms in children associated with the development of obesity that included differences between exposed and non-exposed children in leptin, endothelin-1, glucagon-like peptide-1 (GLP 1), ghrelin, and glucagon [29]. We were unable to include biomarkers in our study and were limited in our exposure assessment since we considered exposure over the previous year to the ENSANUT survey, not capturing chronic nor cumulative exposure. Reconstructing history of exposure, for example, considering mobility, was impossible. Although a life-course assessment and biomarkers of PM_2.5_ exposure in the GMCA would be desirable, the limitations to carry out such a study in a representative sample of the population are considerable.

Several factors known to affect BMI, such as dietary habits, physical activity patterns, sedentary behavior, as well as history of respiratory illnesses (that could have restricted the performance of physical activity), were not available for this study. However, with the exception of physical activity, these are not confounders of the association. We were able to control for SES, which may be considered a surrogate for the omitted predictors, and this may have reduced unmeasured associations from omitted BMI predictors. As noted above, lower SES was associated with higher PM_2.5_ exposure; if SES can be a surrogate for diet quality, PM_2.5_ could be considered an obesogenic if lower SES was associated with lower diet quality; however, higher SES has been previously associated with a lower diet quality in this population [49]. Future studies should include diet quality to better investigate this complex relation. We were able to reproduce the prevalence of overweight and obesity found in other studies using ENSANUT data, showing a decrease in overweight and an increase in obesity between surveys, while normal weight remained similar, suggesting that obesity prevention interventions should particularly target individuals with overweight.

A strength of our research is the use of a state-of-the-art hybrid spatio-temporal model with satellite-derived aerosol optical depth measures to assess PM_2.5_ exposure with higher resolution than previous 10 × 10 Km^2^ estimates employed in similar studies [16,50]. This allowed us to assess exposure to PM_2.5_ with estimates from a novel model that predicts ground-level PM_2.5_ concentration with resolution of 1 × 1 km^2^ for the GMCA. The findings from our study add to the limited evidence on the advantages of using remote-sensing-derived estimates of PM_2.5_ in cities of the developing world that frequently have scarce coverage from ground-level monitoring networks. We used quartiles of PM_2.5_ to identify non-linearities in the exposure–response relationship; however, this approach ignores intra-category variation, which in turn could have reduced our study’s power to detect an association. Thus, future analyses should consider the use of non-linear terms (i.e., splines or fractional polynomials) to address this limitation [51].

Another strength of our study is the representativeness of our population sample; however, survey design sample weighted analysis methods trade estimation precision to obtain minimally biased coefficients that more accurately reflect the characteristics of the population from which the survey sample is drawn compared to coefficients derived from unweighted analysis of the same sample. The trade-off of precision for unbiasedness can be seen in the larger standard errors and confidence intervals of coefficients, due in part to the reduced degrees of freedom available in a design-based model (see supplementary Table S1 for complete models). Less biased coefficients resulting from survey design sample weighted analysis means we can more confidently generalize our results to the entire population.

Finally, regarding our meta-analysis approach, the term is used for two different purposes in epidemiology, the primary being a method for quantitatively summarizing previously published literature. However, when a single study has two groups or components, such as this one, it can be used to describe the method for deriving the overall effect estimate, since the statistical apparatus is identical. We used the inverse variance weighted averages to combine effect estimates between the two different groups. Regarding the different effect estimates in the two different years, random effect methods for combining effect estimates account for that difference. If the difference is greater than would have been expected given the confidence intervals in the two branches of the study, then a random variance component is greater than zero, and incorporating the variance component correctly accounts for the increased uncertainty in estimating the confidence intervals for the overall effect. We did not have a prior hypothesis as to why we would expect a different response in the two years of the study, and therefore we report the overall effect estimate, with correct confidence intervals that incorporate the differences between the two years.

## 5. Conclusions

We found evidence of an association between average past-year PM_2.5_ exposure and increased odds of obesity. Although we were unable to account for chronic PM_2.5_ exposure, mobility, or dietary information, important strengths of this study are the satellite-derived exposure assessment and its representativeness for the Greater Mexico City Area, among the largest cities in Latin America. More studies such as this are recommended in Latin American cities with similar air pollution and obesity conditions.

## Figures and Tables

**Table 1:** Descriptive statistics in population samples for Mexican National Survey of Health and Nutrition (ENSANUT) 2006 and 2012 from the Greater Mexico City Area.

	Ensanut 2006			Ensanut 2012		
Variable	Children (0–9 Years)	Adolescents (10–19 Years)	Adults (≥20 Years)	Children (0–9 Years)	Adolescents (10–19 Years)	Adults (≥20 Years)
Sample size (*n*)	1005	1082	2281	1233	986	2302
Sample representativeness (*N*)	31,00,923	3,565,877	12,695,627	3,552,214	3,636,732	13,760,947
Sex						
Male (%)	50.3	55.3	44.9	51	49.1	45.9
Female (%)	49.7	44.7	55.1	49	50.9	54.1
Age mean (years) ^a^	4.7 ± 2.9	14.4 ± 2.9	42.9 ± 16.4	4.6 ± 2.7	14.3 ± 2.9	44.9 ± 16.3
SES ^b^	−0.16 ± 0.05	−0.03 ± 0.06	−0.16 ± 0.06	0.05 ± 0.10	0.03 ± 0.082	−0.12 ± 0.05
BMI						
Normal (%)	58.45%	58.01%	27.81%	59.02%	56.48%	26.12%
Overweight (%)	20.05%	25.18%	41.45%	17.42%	22.98%	37.34%
Obesity (%)	21.49%	16.81%	30.73%	23.55%	20.53%	35.94%
Average past year PM_2.5_ (µg/m^3^)	25.9 (2.4)			24.8 (1.5)		

^a^ Age in years: mean ± SD. ^b^ SES: zero-centered mean ± SD.

**Table 2:** Association between past year PM_2.5_ (10 μg/m^3^ increase) with obesity in children, adolescents, and adults from the Greater Mexico City Area using data from the 2006 National Nutrition and Health Survey, ENSANUT 2006.

ENSANUT 2006	Children		Adolescents		Adults	
	*n* = 618		*n* = 801		*n* = 1559	
	*n* = 2,529,289		*n* = 3,548,352		*n* = 12,541,729	
	OR	95% CI	OR	95% CI	OR	95% CI
Logistic model *						
Obesity	1.19	(0.47, 3.08)	**3.53**	**(1.45, 8.58)**	1.01	(0.59, 1.73)
Logistic model stratified by sex **						
Male	0.57	(0.17, 1.95)	**4.19**	**(1.24, 14.19)**	1.09	(0.40, 2.91)
Female	2.7	(0.71, 10.21)	2.64	(0.74, 9.44)	0.94	(0.49, 1.79)
*p* interaction	0.07		0.53		0.82	
Multinomial model (RRR) *						
Overweight	1.57	(0.54, 4.56)	1.36	(0.68, 2.73)	0.82	(0.41, 1.62)
Obesity	1.43	(0.53, 3.84)	**3.89**	**(1.51, 10.03)**	0.89	(0.43, 1.85)

* Adjusted for age in quintiles, sex, socioeconomic status (SES), and smoking status. ** Adjusted for age in quintiles, SES, and smoking status. Statistically significant results (*p* <0.05) are highlighted in bold letters.

**Table 3:** Association between past year PM_2.5_ (10 μg/m^3^ increase) with obesity in children, adolescents, and adults from the Greater Mexico City Area using data from the 2012 National Nutrition and Health Survey, ENSANUT-2012.

ENSANUT 2012	Children		Adolescents		Adults	
	*n* = 752		*n* = 718		*n* = 1538	
	*n* = 2,769,354		*n* = 3,245,296		*n* = 11,415,512	
	OR	95% CI	OR	95% CI	OR	95% CI
Logistic model *						
Obesity	1.98	(0.92, 4.22)	**3.79**	**(1.40, 10.24)**	2.73	(0.97, 7.71)
Logistic models stratified by sex **						
Male	2.29	(0.69, 7.55)	2.61	(0.41, 16.73)	2.56	(0.59, 11.20)
Female	1.29	(0.79, 2.10)	7.83	(0.96, 64.12)	2.88	(0.83, 10.04)
*p* interaction		0.44	0.44		0.85	
Multinomial model (RRR) *						
Overweight	0.84	(0.02, 42.96)	2.34	(0.83, 6.65)	0.39	(0.13, 1.18)
Obesity	1.69	(0.63, 4.59)	**4.82**	**(2.16, 10.76)**	1.54	(0.46, 5.14)

* Adjusted for age in quintiles, sex, SES, and smoking status. ** Adjusted for age in quintiles, SES, and smoking status. Statistically significant results (*p* <0.05) are highlighted in bold letters.

## Supplementary Materials

The following are available online at https://www.mdpi.com/1660-4601/18/5/2301/s1: Table S1. Results from crude and weighted models for the association between past year PM2.5 (10 μg/m3 increase) with obesity in children, adolescents and adults* from the Greater Mexico City Area using data from the National Nutrition and Health Surveys, ENSANUT-2006 and 2012.
