# Supplementary material for: Exposure to PM2.5 and Obesity Prevalence in the Greater Mexico City Area
Source: Int J Environ Res Public Health. 2021 Feb 26;18(5):2301. doi: 10.3390/ijerph18052301 (PMC7956483; doi:10.3390/ijerph18052301)
Supplement: Supplementary file 1 [file ijerph-18-02301-s001.pdf]

Supplementary Table S1. Results from crude and weighted models for the association between past year $PM_{2.5}$ (10 μg/m^3 increase) with obesity in children, adolescents and adults* from the Greater Mexico City Area using data from the National Nutrition and Health Surveys, ENSANUT-2006 and 2012.

	**Children**		**Adolescents**		**Adults**	
	**OR**	**95% CI**	**OR**	**95% CI**	**OR**	**95% CI**
ENSANUT 2006						
Weighted model	*n* = 2,529,289		*n* = 3,548,352		*n* = 12,541,729	
	1.24	(0.49, 3.13)	**3.53**	**(1.45, 8.58)**	1.01	(0.59, 1.73)
Crude model	*n* = 618		*n* = 801		*n* = 1559	
	0.95	(0.39, 2.28)	**2.38**	**(1.05, 5.38)**	1.09	(0.66, 1.81)
			**ENSANUT 2012**			
Weighted model	*n* = 2,769,354		*n* = 3,245,296		*n* = 11,415,512	
	1.77	(0.82, 3.86)	**3.79**	**(1.40, 10.24)**	2.73	(0.97, 7.71)
Crude model	*n* = 753		*n* = 758		*n* = 1559	
	1.96	(0.56, 6.77)	2.44	(0.76, 7.84)	**2.16**	**(1.04, 4.48)**

Statistically significant results ($p < 0.05$) are highlighted in bold letters.
